# Spray‐Dried Sodium Zirconate: A Rapid Absorption Powder for CO_2_ Capture with Enhanced Cyclic Stability

**DOI:** 10.1002/cssc.201700046

**Published:** 2017-04-13

**Authors:** Faith Bamiduro, Guozhao Ji, Andy P. Brown, Valerie A. Dupont, Ming Zhao, Steven J. Milne

**Affiliations:** ^1^ School of Chemical and Process Engineering University of Leeds Leeds LS2 9JT United Kingdom; ^2^ School of Environment Tsinghua University Beijing 100084 P. R. China

**Keywords:** absorption, co_2_ capture, kinetic analysis, sodium zirconate, spray drying

## Abstract

Improved powders for capturing CO_2_ at high temperatures are required for H_2_ production using sorption‐enhanced steam reforming. Here, we examine the relationship between particle structure and carbonation rate for two types of Na_2_ZrO_3_ powders. Hollow spray‐dried microgranules with a wall thickness of 100–300 nm corresponding to the dimensions of the primary acetate‐derived particles gave about 75 wt % theoretical CO_2_ conversion after a process‐relevant 5 min exposure to 15 vol % CO_2_. A conventional powder prepared by solid‐state reaction carbonated more slowly, achieving only 50 % conversion owing to a greater proportion of the reaction requiring bulk diffusion through the densely agglomerated particles. The hollow granular structure of the spray‐dried powder was retained postcarbonation but chemical segregation resulted in islands of an amorphous Na‐rich phase (Na_2_CO_3_) within a crystalline ZrO_2_ particle matrix. Despite this phase separation, the reverse reaction to re‐form Na_2_ZrO_3_ could be achieved by heating each powder to 900 °C in N_2_ (no dwell time). This resulted in a very stable multicycle performance in 40 cycle tests using thermogravimetric analysis for both powders. Kinetic analysis of thermogravimetric data showed the carbonation process fits an Avrami–Erofeyev 2 D nucleation and nuclei growth model, consistent with microstructural evidence of a surface‐driven transformation. Thus, we demonstrate that spray drying is a viable processing route to enhance the carbon capture performance of Na_2_ZrO_3_ powder.

## Introduction

Powder sorbents for CO_2_ at high temperatures are of interest for a number of applications, including the production of H_2_ by steam reforming, in which removal of CO_2_ shifts the chemical equilibrium in favor of greater H_2_ yield and purity. Sorption‐enhanced steam reforming (SESR) based on a CaO sorbent (CaO_(s)_+CO_2(g)_⇄CaCO_3(s)_) has been demonstrated at the research level.^1^ Calcium oxide sorbents work best at approximately 600–700 °C, and hence, coupled to steam reforming reactions; the sorbent may be regenerated by calcination in air at around 800 °C or above. This type of calcium looping technology has been considered widely for post‐combustion capture (PCC) from fossil‐fuel‐fired power plants (notably, coal‐fired) and other single‐point industrial emitters.^2^ The technology could be implemented using two parallel, fluidized beds operating as carbonator and regenerator, or using fixed‐bed reactors with alternating carbonation/calcination reactions by feed‐flow control.^2^ For the proposed implementation in PCC, the decarbonation step would be performed in a near pure CO_2_ stream, necessitating calcination temperatures ≥950 °C.^2^ In SESR applications, in which oxygen looping is employed to exchange oxygen with the metal catalyst, air (oxygen depleted) would be the sorbent regeneration stream at temperatures ≥800 °C.^1^

An acceptable sorbent for PCC or SESR should have a high CO_2_‐uptake capacity per unit mass and remain close to its original CO_2_‐capture capacity over repeated carbonation/regeneration cycles.^3^ Material costs should be low and the sorbent should be mechanically robust, as in the case of calcium oxide (CaO) and other inorganic oxides. CaO from limestone is the most inexpensive and readily available options. CaO however shows serious loss of CO_2_ capacity after repeated calcination cycles at 800 °C owing to the effects of partial sintering and loss of surface area and porosity.^4^

A number of additive powders (e.g., SiO_2_, Al_2_O_3_, ZrO_2_) have been investigated as means of improving the multicycle stability of CO_2_‐capture performance of the active CaO component.^3^ The greater the volume fraction of the refractory additive the more durable the sorbent, but there is a trade‐off in the dilution of the active component that leads to loss of initial capture capacity: 20–30 wt % is a common compromise loading. The added oxide component often reacts with CaO to form a binary compound, and it is this compound, for example, Ca_12_Al_14_O_33_ (mayenite), which acts as the “refractory spacer” second phase designed to inhibit CaO particle sintering and densification.3g–3k A uniform distribution of the second phase is essential to minimize densification of the active CaO phase and suppress multicycle degradation. The performance of a range of CaO‐based sorbents is summarized in the study of Zhao et al.3m The more complex (and costly) the processing technique, for example, sol‐gel or chemical templating, the finer the particle size, and the more uniform the dispersion. Consequently, solution‐derived composite powders generally have the best multicycle performance relative to the base CaO sorbent material.

Another approach to avoid multicycle powder densification problems has been to use alternative sorbent materials to CaO, such as Li_4_SiO_4_ and Li_2_ZrO_3_.^5^ The latter has received considerable attention for both post‐ and pre‐combustion capture, and for SESR applications. Li_2_ZrO_3_ absorbs CO_2_ according to the reversible reaction: Li_2_ZrO_3(s)_+CO_2(g)_→Li_2_CO_3(s)_+ZrO_2(s)_ (giving a maximum increase in sorbent mass of 28 %). It also acts as a basic catalyst that has the advantage of promoting tar degradation in SESR processes. However, its utilization has been inhibited by poor reaction kinetics at low CO_2_ partial pressures (<0.02 MPa) and high temperatures (>500 °C). The more active, metastable, tetragonal crystal structure—the major contributor to CO_2_ chemisorption—is potentially transformed to a less reactive monoclinic form during high‐temperature cycling. The Li_2_ZrO_3_‐based sorbents are best suited to processes operating at temperatures <550 °C such as steam reforming of simple compounds such as methane, ethanol, or glycerol. Solid solutions of Li_2_ZrO_3_ with Na_2_ZrO_3_ have also received attention.^6^

There are also reports on the use of Na_2_ZrO_3_ and K_2_ZrO_3_ as CO_2_ sorbents.^7^ From thermodynamic considerations, Na_2_ZrO_3_ and K_2_ZrO_3_ absorb CO_2_ at lower CO_2_ partial pressures and higher temperatures than Li_2_ZrO_3_. However K_2_ZrO_3_ sorbents are more difficult to regenerate. To reach a good balance between ease of capture and regeneration at high temperatures (≈650–750 °C), Na_2_ZrO_3_ is more promising than either Li_2_ZrO_3_ or K_2_ZrO_3_.

The CO_2_ uptake and regeneration of Na_2_ZrO_3_ proceed according to the reversible reaction: Na_2_ZrO_3(s)_+CO_2(g)_→Na_2_CO_3_(s)+ZrO_2_(s). Conventionally, Na_2_ZrO_3_ sorbents are synthesized by a SS reaction between Na_2_CO_3_ and ZrO_2_ at high temperatures (≥1000 °C), resulting in large (micrometer) agglomerated particles, with long diffusion paths for subsequent carbonation.

To reduce the particle size of Na_2_ZrO_3_ sorbents, a number of solution‐based synthesis routes were developed.7a, 8 These result in faster carbonation rates since a greater proportion of the CO_2_ uptake occurs through interfacial solid–gas reactions, and the diffusion lengths for ion migration in the later stages of the reaction (in which the rate of mass transfer is controlled by SS diffusion) are reduced.

Herein, we use scanning and transmission electron microscopy with energy‐dispersive analysis of X‐rays (EDX) to investigate the microstructural differences between Na_2_ZrO_3_ particles produced by spray drying a mixed acetate solution, and powders prepared by conventional SS reaction. The structural differences we identify account for much faster rates of carbonation in spray‐dried (SD) forms. A CO_2_ conversion of approximately 0.18 g${}_{\mathrm{CO}{}_{2}}$ g-1sorbent (≈75 % of theoretical capacity) is demonstrated for the SD powder after only 5 min exposure to 15 vol % CO_2_ at 700 °C, namely, under carbonation conditions pertinent to SESR. Stable multicycle performance is demonstrated for both powder types over a 40 cycle thermogravimetric testing program (decarbonation at 900 °C) but because of the slower rate of carbonation for the conventionally prepared Na_2_ZrO_3_, its conversion is only about 50 % of the theoretical capacity under these conditions (which are relevant to implementation in SESR). Finally, we link our microstructural observations to kinetic modelling of the CO_2_‐absorption profiles measured during carbonation to gain mechanistic insights into the surface‐driven absorption process.

## Results and Discussion

### Phase analysis and particle structure: as‐prepared powders

**Figure 1:**
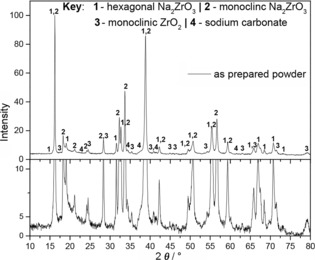
XRD pattern for the Na_2_ZrO_3_ SD powder (top figure) with indexing to a mixture of the hexagonal and monoclinic phases. An expanded intensity scale in the lower figure aids the identification of small quantities of residual intermediate Na_2_CO_3_ and ZrO_2_ phase.

X‐ray diffraction (XRD) patterns confirmed that both SD and solid‐state (SS) powders contained crystalline Na_2_ZrO_3_, in the form of hexagonal and monoclinic polymorphs. Figure 1 presents the XRD pattern for the SD powder. Minor peaks of ZrO_2_ (monoclinic) and very weak peaks of Na_2_CO_3_ were detected, consistent with residual intermediate phases from the following reaction [Reaction (R(R1), only inorganic products are represented]:

(R1) $$2\;\mathrm{Na}\left(\mathrm{CH}{}_{3}\mathrm{COO}\right)+\mathrm{Zr}\left(\mathrm{CH}{}_{3}\mathrm{COO}\right){}_{4}\to \mathrm{Na}{}_{2}\mathrm{CO}{}_{3}+\mathrm{ZrO}{}_{2}\to \mathrm{Na}{}_{2}\mathrm{ZrO}{}_{3}+\mathrm{CO}{}_{2}$$

The very weak Na_2_CO_3_ peaks relative to the XRD peaks for ZrO_2_ are consistent with the former being poorly crystallized. The conventional SS powder gave similar diffraction patterns to the SD material (Figure S1 in the Supporting Information).

**Figure 2:**
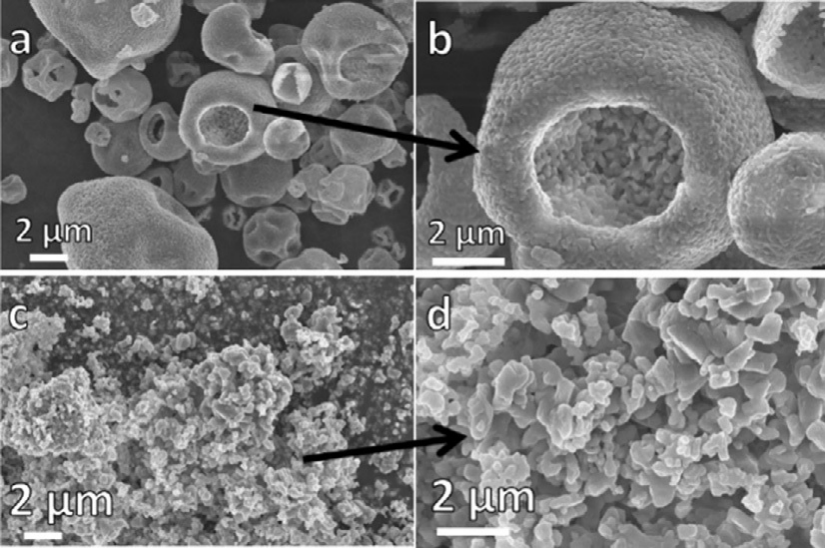
a, b) SEM images of the SD sorbent powders showing partially deflated, hollow granules 1–10 μm in size with a substructure composed of 100–300 nm primary particles; c, d) conventional powder prepared by SS reaction with solid agglomerates 10 s μm in size and primary particle size ≈1 μm.

**Figure 3:**
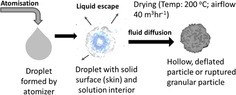
Schematic of Na_2_ZrO_3_ particle formation during spray drying showing the likely formation route of hollow, ruptured granules; the released liquid goes on to form smaller secondary granules.

SEM (Figure 2) revealed the SD powders to be hollow, perforated, and partially collapsed spherical granules. These ranged in size from 1–10 μm (Figure 2 a). The walls of the granules were composed of interlocking primary particles (100–300 nm in size, Figure 2 b) and were a single particle in thickness (the 100–300 nm wall thickness is illustrated in Figure S2 in the Supporting Information). We have observed similar particle structures previously, for example, in ZrO_2_ granules that were spray dried from acetate solution.^9^ This type of structure is consistent with a formation mechanism in which liquid atomized droplets, upon entering the heated chamber of the spray dryer, first develop a solid, pliable surface skin of salt particles that surrounds a liquid core. After continued heating, pressure builds up and is released by bursting of the outer solid skin, resulting in characteristic surface rupturing of the hollow granule. If the outer skin remains pliable at this stage, the walls collapse to create deformed, hollow spheres. The expelled liquid from the interior of the droplet forms a secondary aerosol, which results in a series of smaller granules. A schematic of the proposed SD granule formation mechanism is shown in Figure 3.

The SS powders were composed of densely agglomerated granules, tenths of μm in size, typical of a conventionally prepared mixed‐oxide ceramic powder; primary particle size was approximately 0.05–1 μm (Figure 2 c, d).

### Carbonation characteristics and effect on particle structure

**Figure 4:**
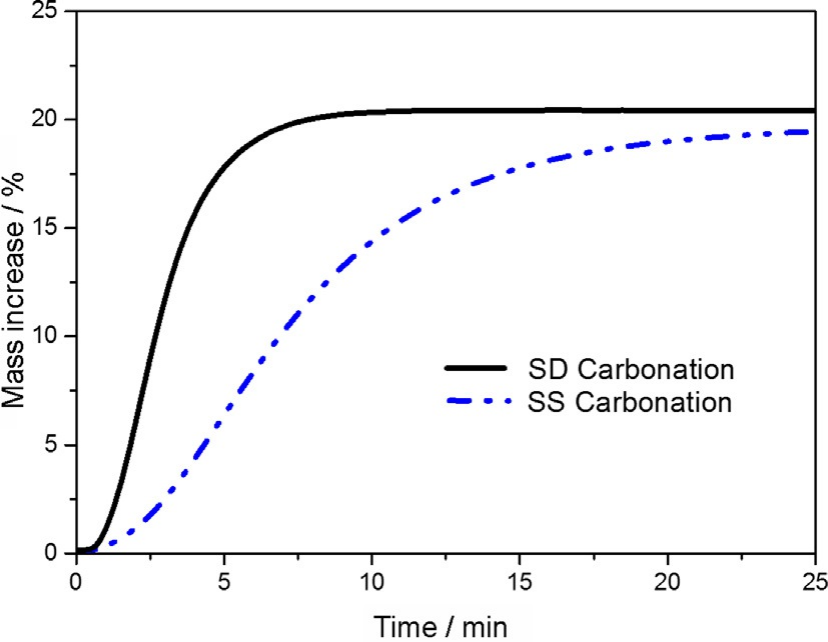
TGA profiles of SD and SS powders carbonated under isothermal conditions (700 °C, 15 % CO_2_).

To assess the baseline CO_2_‐uptake performance of the SD and SS powders, the response to prolonged exposure to 15 % CO_2_ at 700 °C was analyzed (Figure 4). The SD powders reached a steady‐state increase in mass after about 10 min, equivalent to 0.20 g${}_{\mathrm{CO}{}_{2}}$ g-1sorbent uptake and a molar conversion of approximately 85 % of theoretical capacity. After 5 min, the uptake was about 0.18 g${}_{\mathrm{CO}{}_{2}}$ g-1sorbent . The SS powder approached a similar steady‐state level of carbonation but required a dwell period of almost 25 min as opposed to only 10 min for the SD powder, indicating a much slower rate of carbonation in the conventional SS powder.

**Figure 5:**
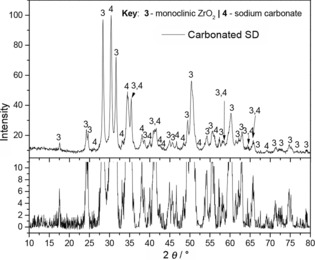
XRD pattern of granules obtained after extended carbonation of the SD sorbent (25 min carbonation) revealing the presence of a mixture of ZrO_2_ and Na_2_CO_3_ carbonation products with no other phases detected.

The XRD patterns of both powders collected after the end of the isothermal thermogravimetric analysis (TGA) experiment were similar. The pattern for the SD powder is shown in Figure 5, indicating a mixture of Na_2_CO_3_ and ZrO_2_, with no evidence of unreacted Na_2_ZrO_3_ (Figure 5). The carbonated SS powder pattern is shown in the Supporting Information. This confirms the carbonation reaction of the Na_2_ZrO_3_ phase contained in the calcined starting powder [Reaction (R(R2)] had reached completion (subject to XRD detection limits).

(R2) $$\mathrm{Na}{}_{2}\mathrm{ZrO}{}_{3}+\mathrm{CO}{}_{2}\to \mathrm{Na}{}_{2}\mathrm{CO}{}_{3}+\mathrm{ZrO}{}_{2}$$

**Figure 6:**
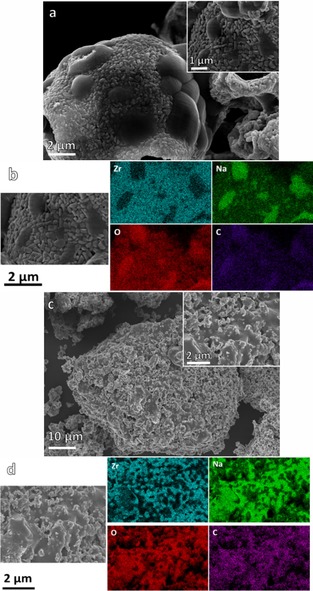
a) SEM image of SD granules obtained after extended carbonation (25 min 15 % CO_2_ at 700 °C); b) EDX mapping of the surface of the SD granule; c) conventional SS powder after carbonation; d) EDX mapping of the surface of the SS agglomerate.

The SEM images of the powders produced after 25 min isothermal carbonation revealed the carbonated SD granules retained the general structure of the as‐prepared material Figure 6 a (compared to Figure 2 b); likewise, there was little change in the general form of the SS agglomerates (Figure 6 c compared to Figure 2 c). The surface of the carbonated SD granules revealed localized pockets with a smooth, glass‐like appearance.

Close inspection indicated that a similar phase was also interspersed within the interlocking submicron particles that made up the remainder of the granule surface (Figure 6 a). The SEM/EDX elemental maps indicated the smooth regions to be Na‐rich (Figure 6 b), and therefore, we attribute these to be Na_2_CO_3_, which under the carbonation conditions employed had softened and flowed into isolated islands. The remainder of the carbonated‐granule structure was made up of interlocking faceted particles that were Zr‐rich (Figure 6 b)—these are assumed to be the ZrO_2_ phase identified by XRD. There was also some localized glass‐like phase interspersed within the (crystalline) ZrO_2_ particles. The SS agglomerates showed similar evidence of a glass‐like Na‐rich phase surrounding Zr‐rich particulate material (Figure 6 d).

### Multicycle carbonation/decarbonation performance

**Figure 7:**
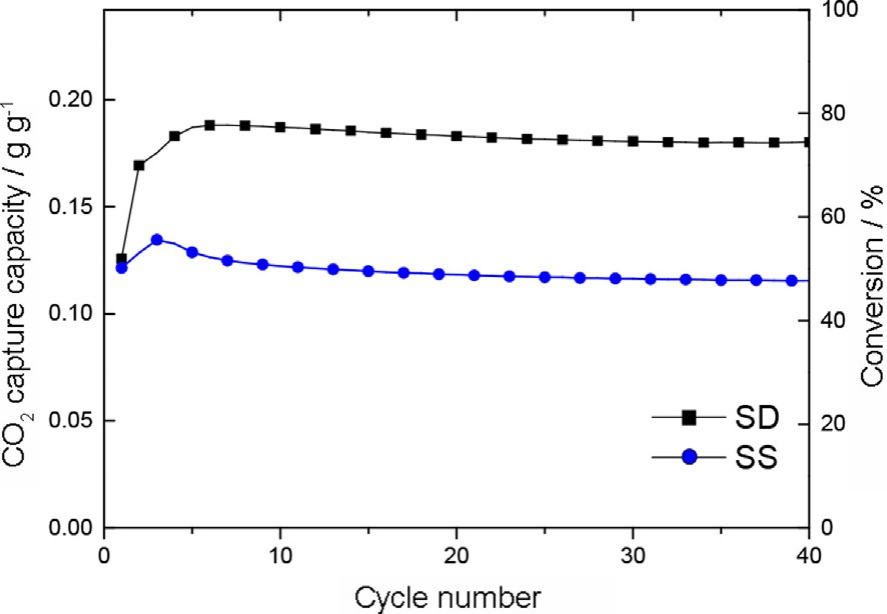
Carbonation performance for SD and SS powders (carbonation 5 min in 15 % CO_2_ at 700 °C, regeneration by increasing temperature to 900 °C at 20 °C min^−1^ in N_2_).

The multicycle performance of the SD powder and the conventional powder over 40 TGA carbonation/decarbonation cycles, is summarized in Figure 7 (multicycle TGA plots are shown in the Supporting Information). An increase in the level of CO_2_ uptake was observed over the first 3 cycles for each powder; this type of self‐activation has been observed for other oxide sorbent powders, for example, CaO, and can be attributed to the generation of porosity in the powder owing to outgassing in the first few decarbonation cycles.^10^ After the initial selfactivation period, the uptake capacity of both the SD and SS powders showed a remarkable stability, indicating high durability to be an intrinsic feature of Na_2_ZrO_3_ sorbents (as discussed below). The variation in mass conversion of the SD powder was <5 % between cycles number 3 and 40. The CO_2_‐uptake level was approximately 0.18 g${}_{\mathrm{CO}{}_{2}}$ g-1sorbent (4.1 mmol g^−1^) in cycle 4 corresponding to a molar conversion efficiency of about 75 %. Because of the slower rate of carbonation of the SS powder (as identified in Figure 4) the level of uptake after the set 5 min carbonation within multicycle experiments was lower, 0.12 g${}_{\mathrm{CO}{}_{2}}$ g-1sorbent (2.7 mmol g^−1^) or about 50 % conversion by mass under multicycle conditions.

**Figure 8:**
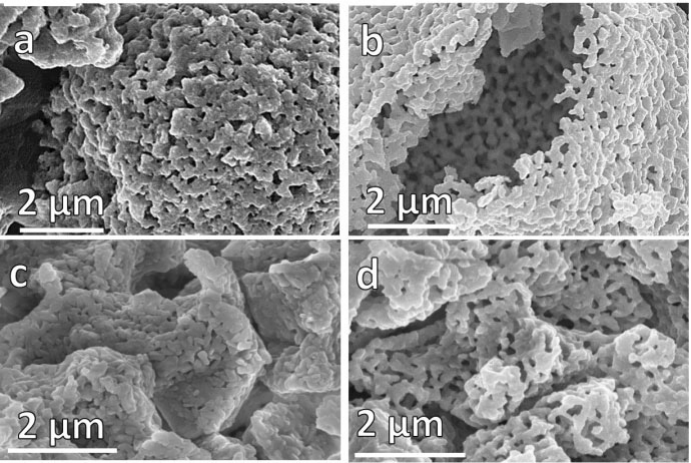
SEM images showing the morphology and surface structure of SD granules after multicycle TGA ending on carbonation: a) 10 cycles and b) 30 cycles. Corresponding images of the SS powders are shown in c) and d).

SEM micrographs showed the particle structure of decarbonated SD and SS powders after 10 and 30 TGA cycles, indicating a more porous structure (Figure 8 a, b) than for the asprepared example (Figure 2). This is consistent with reports for other oxide sorbents for which an initial increase in porosity owing to self‐activation associated with the first few carbonation/decarbonation cycles is shown.^10^, ^11^ The cycled SS powders were also more porous than the as‐prepared SS samples (Figure 8 c,d).

### TEM of spray‐dried powder

**Figure 9:**
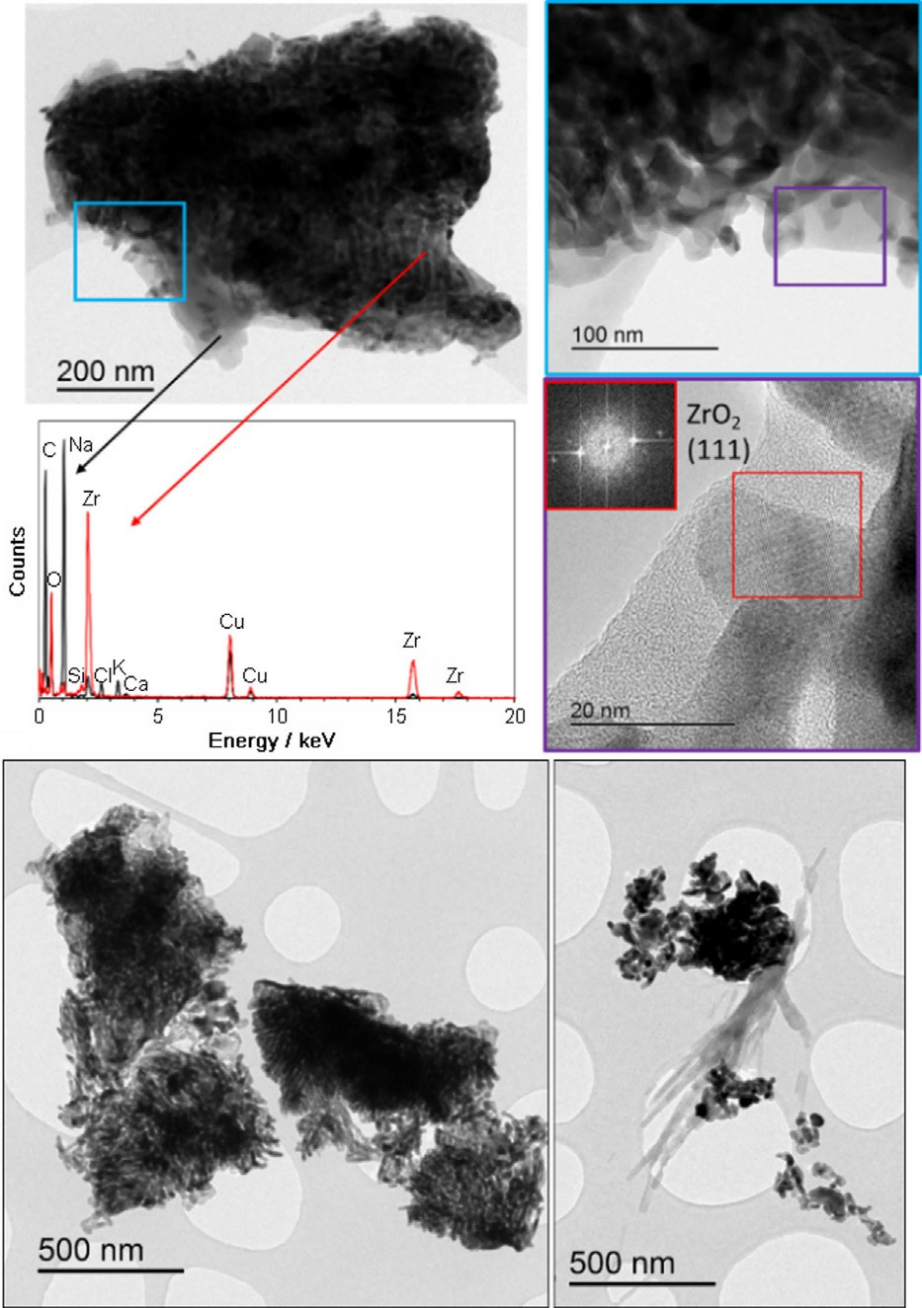
a) TEM of the carbonated SD powder after one TGA cycle and dispersion in heptane. Top left, bright field TEM image of a granule fragment that has a polycrystalline substructure, as highlighted by the high magnification inset (top right, blue box). Lattice imaging of this region is shown in the bottom right image (purple box) and inset shows fast Fourier Transform. EDX spectra (bottom left) of two different regions of the particle: the polycrystalline network regions (red) are Zr and O rich whereas the glassy region is Na and C rich (the background Cu signal is from the support grid).(b) TEM images of carbonated SD granule fragments after one TGA cycle (left hand image) and 20 TGA cycles (right hand image) following dispersion in acetone.

Analysis by TEM of the carbonated SD powder after one TGA cycle and dispersion in heptane is shown in Figure 9 a. Only fragments of the granules could be imaged as full‐size granules are not electron transparent. The fragment shows a polycrystalline substructure (top right image in Figure 9 a). Lattice imaging of this region reveals crystalline particles in a glassy matrix (bottom right image) with fast Fourier Transform (inset) showing the lattice spacing of the particle identified in the red box to be 2.89 nm, consistent with the ZrO_2_ (111) spacing (ICDD ref. file 00‐037‐1484). EDX spectra (bottom left, Figure 9 a) show that the polycrystalline regions (red) are Zr and O rich whereas EDX spectra of the glassy regions (black) are Na and C rich, consistent with Na_2_CO_3_ (the background Cu signal is from the support grid). These findings are in agreement with the information inferred by SEM/EDX of full‐size SD granules imaged following extended carbonation experiments (Figure 6) and confirm that the walls of the hollow granules are composed of a network of interlocking submicrometer, crystalline ZrO_2_ particles with regions of partially glassy Na_2_CO_3_ phase interspersed between them (only partially glassy because XRD identifies a minor amount of crystalline Na_2_CO_3_).

To reveal more information on the spatial distribution of the component phases, two other TEM samples were prepared: a sample collected after one TGA cycle, the other after 20 cycles. This time powders were dispersed in acetone instead of heptane. Acetone is a polar solvent in which Na_2_CO_3_ and any hydroxyl–carbonate phases that may form upon storage in air, or on exposure to moisture present in dispersant liquids (e.g., bicarbonate), are soluble and leach out of the granule fragments. TEM showed that the acetone‐dried samples were indeed more porous (Figure 9 b), suggesting that the soluble (Na_2_CO_3_) material had originally been located between the ZrO_2_ nanoparticle networks, corroborating the interpretations of SEM images (which showed glassy material amongst ZrO_2_ particles in addition to segregated pockets of Na_2_CO_3_). In some areas of the 20 cycle image, the leached carbonate phase has re‐precipitated in an acicular morphology.

### Carbonation reaction: kinetic analysis

A set of isothermal TGA carbonation experiments were designed to identify the reaction model that best describes the carbonation process of the SD and SS powders and to derive apparent kinetic parameters.

Conversion [Eq. (1)] was calculated by finding the minimum and maximum measured TGA masses over the cycle step considered (a cycle consisting of carbonation followed by calcination). For carbonation, the minimum mass is the initial mass at *t=*0 (*m*_0_), whereas the maximum is the final mass at *t*=*t*_f_ (*m*_f_).

(1) $$\alpha \left(t\right)=\frac{m\left(t\right)-m_{0}}{m_{f}-m_{0}}$$

Conversion versus time data (*α* vs. *t*) can then be represented using several models of SS (gas) reactions. Hancock and Sharp's method^12^ assigns a model or a family of models according to the value of *m*, as defined in Equation (2):

(2) ln[-ln(1-α)]=mlnt+lnB

in which *B* is a constant, the conversion values (*α*) range typically between 0 and 0.5, and plotting ln[−ln(1−α)] versus ln *t* produces a straight line fit with gradient *m*.

**Figure 10:**
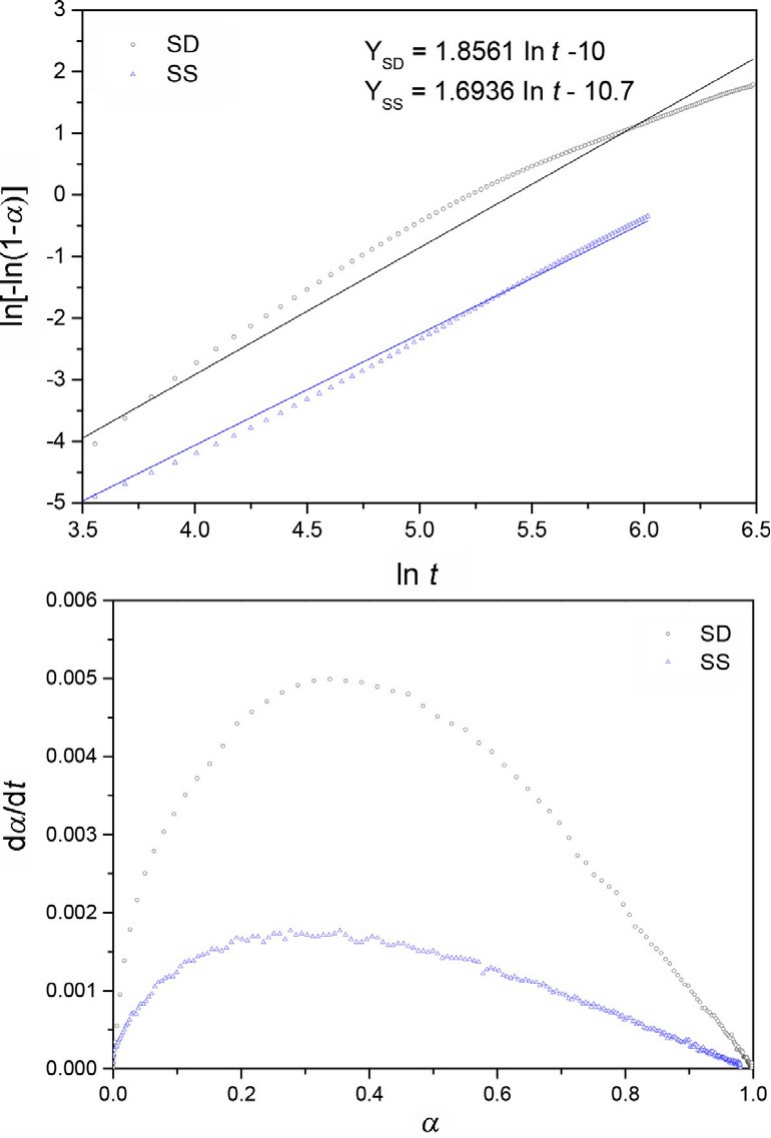
Model identification for carbonation conversion factors of SD and SS powders using a) Hancock and Sharp method,^12^ indicating linear fit with gradient *m≈2* [Eq. (2)] corresponding to Avrami–Erofeyev A2 model; b) Khawam and Flannagan method,^13^ indicating dome‐shape of d*α*/d*t* vs. *α*.

Figure 10 a shows the linear fit for the SD and SS powders carbonating at 700 °C with best fit values of *m* and ln *B*.

According to Hancock and Sharp,^12^ the SD and SS powders exhibited *m* values of 1.86 and 1.69 respectively, both corresponding to Avrami–Erofeyev (also known as JMAEK) models close to *m*=2, termed A2 models. Avrami–Erofeyev A*N* models, with values of *N*≥1, are known as “nucleation and nuclei growth models”. In the case of the carbonation of the SD and SS Na_2_ZrO_3_ crystals, with fitted values of *m* of 1.9 and 1.7, both close to *N*=2, disc‐like are the most likely nuclei shapes.

Further confirmation of the Avrami–Erofeyev model being identified as best fitting the SD and SS Na_2_ZrO_3_ carbonation reactions is found using the method described by Khawam and Flanagan.^13^ In this method, the shape of the plot d*α*/d*t* versus *α* is used to determine the most likely reaction model, with Avrami–Erofeyev displaying a unique dome‐like profile with the apex located at *α* values between 0.3 and 0.4 for model A2. Figure 10 b shows that the carbonation of both the SD and SS Na_2_ZrO_3_ powders exhibited dome shapes with apices between 0.3 and 0.4, corresponding roughly to the A2 model.

Reaction kinetics of the Avrami–Erofeyev A*N* models can be described by the equation relating the integral‐conversion function (*g*(*α*)) to the reaction time following Equation (3):

(3) $$g\left(\alpha \right)=k\times t=\left(-\ln\left(1-\alpha \right)\right){}^{1/N}$$

in which the rate constant *k* typically follows Arrhenius’ law [Eq. (4)]:

(4) k=A×exp(-E/RT)

in which *A* is the pre‐exponential factor, *E* is the activation energy, *R* is the universal gas constant, and *T* is the temperature in K.

Here, the carbonation having been performed at 700 °C, one value of *k* was obtained for each of the materials tested (SD and SS Na_2_ZrO_3_ powders).

Inverting Equations (1) and (3) allows the calculation of a modeled value of mass increase (in %) as function of time according to Equations (5), (6):

(5) $${\mathrm{Mass}\;\;\mathrm{increase}}_{\mathrm{model}}\left[\%\right]=100\times \frac{m\left(t\right)-m_{0}}{m_{0}}=\alpha \times \frac{\left(m_{f}-m{}_{0}\right)}{m_{0}}$$

(6) $$\mathrm{With}\;\;\alpha =1-\exp\left[-\left({\left(kt\right)}^{N}\right)\right]$$

**Figure 11:**
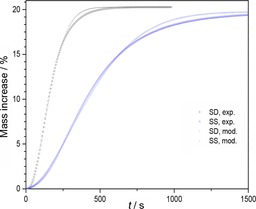
Time profiles of mass increase from experimental TGA compared with modeled mass increases for SD and SS powders using Equations (5) and (6) with parameters *N*_SD_=1.856, *k*_SD_=5.400×10^−3^ s^−1^, *m*_0, SD_=11.07 mg, *m*_f, SD_=13.32 mg, *N*_SS_=1.693, *k*_SS_=1.971×10^−3^ s^−1^, *m*_0, SS_=11.

Figure 11 compares the experimentally obtained % mass increases versus time of the SD and SS Na_2_ZrO_3_ powders during carbonation at 700 °C with their modeled counterpart using Equations (5) and (6), and provides a final test of the suitability of the chosen models with their derived kinetic rates. It can be seen that an excellent match between experimental and modeled mass increases was obtained for both materials.

Both modelling methods indicate 2 D nucleation and nuclei growth for the carbonate phase, which when combined with the SEM observations (Figures 2 and 6) suggest a surface‐driven transformation of the Na_2_ZrO_3_ granules, consistent with a porous Na_2_CO_3_ and ZrO_2_ surface layer discussed in another Na_2_ZrO_3_ study.7d

In summary, SD Na_2_ZrO_3_ granules exhibit rapid CO_2_ uptake reaching 0.18 g${}_{\mathrm{CO}{}_{2}}$ g-1sorbent within only 5 min (15 % CO_2_ at 700 °C), some 50 % greater conversion within this process‐relevant time period than the conventionally prepared SS powder. Both powder types are highly durable, showing minimal decay (<5 %) in uptake capacity after the 40 cycles test under conditions relevant to steam reforming.

**Figure 12:**
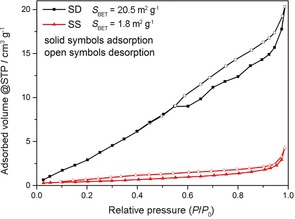
N_2_ adsorption/desorption isotherms for the Na_2_ZrO_3_ sorbent powders produced by spray drying and conventional SS reaction. STP: standard temperature and pressure conditions.

Thus, we demonstrate the intrinsically superior durability of Na_2_ZrO_3_, and that the rate of carbonation may be improved through simple spray drying, which is an industrially scalable process that provides a fine primary particle size within a porous granular structure. Confirmation of a higher surface area in the SD powders, as suggested by the SEM images, was obtained from N_2_‐adsorption isotherms (Figure 12). The BET (Brunauer–Emmett–Teller) surface areas of the SD powder were ≈20 m^2^ g^−1^ compared to only ≈2 m^2^ g^−1^ for the SS powder. Hysteresis in the isotherms indicates mesoporosity. Pore volumes measured by the Barrett–Joyner–Halenda (BJH) method were ≈0.039 cm^3^ g^−1^ for the SD powder and 0.007 cm^3^ g^−1^ for the SS powder. This difference is consistent with SEM observations of hollow‐perforated microgranules in SD powders, and dense agglomerates in SS powders. The hollow and perforated microstructure of the SD granules provides easy access of CO_2_ to the inner and outer surfaces of the granule walls. This, allied to the thin wall dimensions, results in a higher proportion of the carbonation process involving a rapid gas–solid reaction (the linear segment of the TGA profile) than is the case for the densely agglomerated SS Na_2_ZrO_3_ powder.

**Figure 13:**
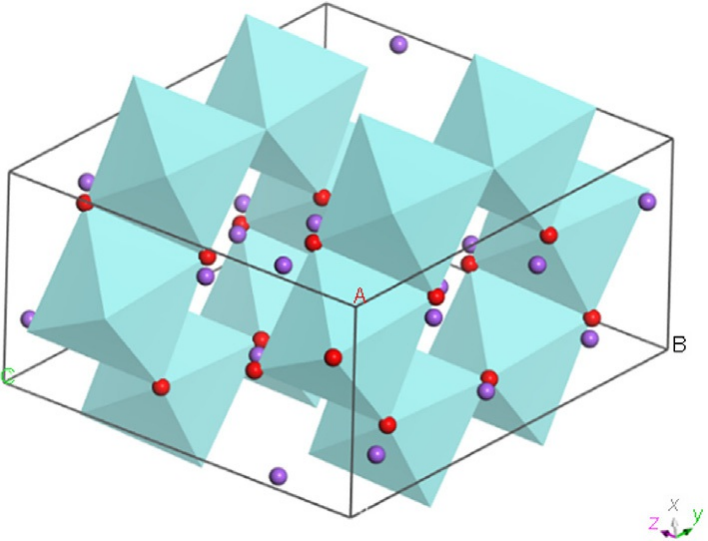
Crystal structure of Na_2_ZrO_3_ (monoclinic): pale blue polyhedral represent edge‐sharing ZrO_6_ structural units; red dots are oxygens and purple dots are sodium ions.

Crystalline Na_2_ZrO_3_ naturally possesses lattice‐scale intimate mixing of refractory ZrO_2_ and active Na_2_O constituents. The crystal structure of the monoclinic form is represented in Figure 13. The lattice‐scale distributions of each component represents ideal mixing of a composite metal‐oxide sorbent material suited to high‐temperature operation, and account for the remarkable durability of Na_2_CO_3_ in multicycle operation. This scale of mixing cannot be achieved by mechanical mixing or chemical precipitation of two‐phase sorbent and refractory spacer powders.

During carbonation, the Na_2_ZrO_3_ crystal lattice decomposes in a surface‐driven process to a truly nanoscale composite of ZrO_2_ and Na_2_CO_3_. From SEM and TEM examination of the walls of the hollow SD granules, a poorly crystallized/glassy carbonate phase segregates. The reverse reaction to regenerate crystalline Na_2_ZrO_3_ occurs readily during the temperature and gas‐swing decarbonation step, once again creating a sorbent with ideal crystal lattice scale distributions of “active” and “spacer” components ready for the next carbonation step. The net result is a very durable single‐phase high‐temperature sorbent.

As mentioned in the Introduction, there is a wide literature on other high‐temperature powder sorbents for CO_2_ capture, most notably for CaO powders in which refractory additives are introduced, for example, ZrO_2_ 3m, 14 to suppress the natural densification (partial sintering) and loss of porosity that degrades cycle on cycle the CaO performance, as outlined in the Introduction. Often, very complex chemical solution precipitation reactions are employed to promote adequate mixing of the two components. In 2012, we proposed a temperature‐induced volume‐expanding phase change additive to disrupt densification,^15^ others later adopted this concept.^16^ However, we found that the volume expansion that occurred between regeneration and carbonation was accommodated in the residual pore spaces and did not induce microcracking to open up porosity prior to the next carbonation step. All of these second‐phase additives to a sorbent powder require complicated processing to achieve significant improvements in durability as performance is only improved if there is intimate mixing of “refractory” additive and sorbent. Even the best chemical or mechanical synthetic processes only give mixing of the two particle types on the submicron scale.

There are a number of literature reports of Na_2_ZrO_3_ as a sorbent for CO_2_: the conditions used for sorption/desorption vary between the different publications. Martínez‐dlCruz and Pfeiffer7d, 7e prepared Na_2_ZrO_3_ by a similar SS route to our SS powder but with calcination at 850 °C for 6 h and found that addition of 20 % excess Na_2_ZrO_3_ produced a phase‐pure product (by XRD). The surface area of this product was approximately 3 m^2^ g^−1^, comparable to the surface area of the SS powder presented herein. Their 20 cycle sorption/desorption studies were conducted in 100 % CO_2_ (as opposed to 15 % herein): temperatures between 550 and 700 °C were found to give the highest uptakes; desorption in N_2_ was conducted at ≤800 °C. Sorption‐dwell times of 30 minutes were adopted, the samples exhibited CO_2_ uptakes corresponding to 18.5–19 mass %.7d, 7e Our SS powder exhibited similar uptake after similar total time periods to these reports (Figure 4) but we adopted a shorter (5 minute) carbonation period in multicycle TGA as this replicated more closely the conditions of a working sorbent. The same group studied the microstructure of their SS powders and concluded that a mesoporous structure was formed on the surface of the agglomerates at sorption temperatures of 300–550 °C, but sintering of this shell layer at temperatures above 550 °C eliminated the porosity and at that stage sorption kinetics were controlled by diffusion processes through a dense Na_2_CO_3_+ZrO_2_ shell.7d This is consistent with our TEM analysis. The effect of relative humidity on the carbonation and decarbonation processes at low temperatures (30–80 °C) for powders produced by SS reaction shows that high humidity has a positive effect, which was attributed to bicarbonate formation at the surface.^17^

Several solution routes have been used to produce Na_2_ZrO_3_ sorbent powders. This includes simple evaporation of sodium acetate and zirconium acetyl acetonate in ethanol and uptakes of CO_2_ of about 21 wt % by TGA over four cycles were recorded involving sorption in 80 % CO_2_ at 600 °C (for >100 minutes) and regeneration in argon at 800 °C.7a, 8 Spray drying of these precursor solutions was also investigated.^8^ Unlike the SD granules of the present work, their spherical granules disintegrated on calcination to produce a nanosized powder of similar particle sizes (≈50 nm) to the powders produced by simple evaporation drying. Hence, both SD and simple evaporation powders within the study of Zhao et al.^8^ exhibited similar CO_2_‐capture properties, achieving around 17.5 wt % mass increase after 200 s in 100 % CO_2_ at 575 °C. Multicycle performance in 50 % CO_2_ up to 11 cycles indicated an uptake of almost 15 wt %.^8^ Sodium oxalate and zirconium nitrate, sodium citrate and zirconyly nitrate aqueous solutions as well as sodium acetate and zirconyl chloride solutions have been used to produce Na_2_ZrO_3_ in an evaporation/drying/calcination process reported by Ji et al.^18^ and Memon et al.^19^

The CO_2_‐capture kinetics of our SD powders compare favorably to other Na_2_ZrO_3_ sorbent powders, although performance comparisons between different laboratories is complicated by the variability in sorption and desorption conditions employed. We demonstrate distinctive microstructural features that lead to high surface areas, which explain the reasons for the characteristic rapid rates of carbonation. The direct like‐for‐like comparison to SS tested under identical TGA conditions provides an unequivocal demonstration of the superior performance of SD. For comparisons with other alkaline metal or alkaline‐earth ceramic sorbents, the reader is directed to a comprehensive review article by Memon et al.^19^

## Conclusions

The microstructural reasons for faster rates of CO_2_ capture by spray‐dried (SD) granules of Na_2_ZrO_3_ relative to a powder prepared by conventional solid‐state (SS) synthesis method have been established using a combination of scanning and transmission electron microscopy, surface area measurements, and kinetic modeling. The hollow and perforated granular structure of SD powders presents a higher surface area than the densely agglomerated conventional powder and promotes the surface‐driven carbonation reaction. This permitted about 75 % of theoretical mass conversion within 5 min exposure to 15 % CO_2_ at 700 °C, compared to only around 50 % for the benchmark conventional SS powder. Although segregation of Na_2_CO_3_ and ZrO_2_ occurs during carbonation, crystalline Na_2_ZrO_3_ is reformed by heating to 900 °C and immediately cooling, ready for the next carbonation step in a multicycle sorption/desorption process. High multicycle durability is an intrinsic feature of Na_2_ZrO_3_ as the active soda component is held within a stable crystal structure. This contrasts to alternative high‐temperature sorbents such as CaO‐based materials in which sintering degrades durability.

## Experimental Section

SD powders were prepared from a starting solution produced by dissolving Na(CH_3_COO)⋅3 H_2_O) (50 mmol) and Zr(CH_3_COO)_2_ (25 mmol) in dilute nitric acid (300 mL) (Sigma–Aldrich reagents) to form a clear solution. This solution was spray dried using a bench‐top spray dryer (SD‐05 Lab‐Plant, UK). The operation conditions were: inlet temperature 200 °C, aspirating air flow at 40 m^3^ h^−1^, peristaltic pump speed 0.6 dm^3^ h^−1^, and compressor pressure of 0.18 MPa. Collected powders were calcined in a box furnace at 900 °C for 2 h to promote formation of Na_2_ZrO_3._ The conventional SS powder was prepared by ball‐milling Na_2_CO_3_ (Acros Organics) and ZrO_2_ (Dynamic Ceramics) powders for 16 h, followed by calcination at 900 °C for 2 h. Nitrogen adsorption/desorption isotherms were measured using a Quantachrome Instruments Nova 2200: surface areas were measured by the BET method and pore volumes by the BJH method. Samples were outgassed under vacuum at 200 °C for 3 h prior to analysis.

The first assessment of the carbonation characteristics of the SD and SS powders involved isothermal TGA in which a ≈15 mg sample was exposed to CO_2_ at 700 °C (Mettler Toledo star 1 TGA/DSC). The sample was first heated to 900 °C (20 °C min^−1^) in N_2_ to remove any traces of hydrated/carbonated surface phases formed during storage. After cooling (20 °C min^−1^) to 700 °C, the gas was switched to 15 % CO_2_/85 % N_2_ and held at this condition for 25 min. Multicycle performance up to 40 cycles was evaluated using 700 °C, 15 % CO_2_/5 min carbonation and regeneration (desorption) achieved by switching to N_2_ and heating at 20 °C min^−1^ to 900 °C and immediately cooling at 20 °C min^−1^ to 700 °C.

XRD data were collected using a Bruker D8 diffractometer (CuK_α_ *λ*=1.5416 Å). Owing to the small quantities of powders generated in the TGA experiments, the powders were deposited on a silicon sample holder. The resulting XRD patterns were analyzed using X′Pert HighScore Plus software (Version 3.0e). The diffraction patterns were compared to standard patterns in the ICDD PDF4 database (International Center for Diffraction Data).

The microstructures of as‐prepared powders, carbonated powders, and powders after multiple carbonation/decarbonation cycles were characterized by using SEM with energy dispersive EDX elemental analysis (LEO 1530 Gemini field emission gun, FEG‐SEM). All samples for SEM were sputter‐coated with a layer of platinum, ≈5 nm in thickness. TEM was used to analyze an SD sample after 1 and 20 successive TGA cycles, ending on a carbonation step (Philips CM200 Field emission gun TEM/STEM with Supertwin Objective lens, and an Oxford Instruments SD 80 mm^2^ X‐max EDX system running INCA software). Powders were prepared for TEM by dispersing in either acetone or heptane (as detailed) and drop‐casting onto standard holey carbon films supported on copper grids (Agar Scientific Ltd).

## Conflict of interest

*The authors declare no conflict of interest*.

## Supporting information

As a service to our authors and readers, this journal provides supporting information supplied by the authors. Such materials are peer reviewed and may be re‐organized for online delivery, but are not copy‐edited or typeset. Technical support issues arising from supporting information (other than missing files) should be addressed to the authors.

Supplementary

## References

[1a] E. J. Anthony , E. M. Bulewicz , L. Jia , Prog. Energy Combust. Sci. 2007, 33, 171–210;

[1b] B. Dou , V. Dupont , P. T. Williams , H. Chen , Y. Ding , Bioresour. Technol. 2009, 100, 2613–2620. 19167215 10.1016/j.biortech.2008.11.037

[2] J. C. Abanades , E. J. Anthony , J. Wang , J. E. Oakey , Environ. Sci. Technol. 2005, 39, 2861–2866. 15884387 10.1021/es0496221

[3a] V. Manovic , E. J. Anthony , Energy Fuels 2010, 24, 5790–5796;

[3b] N. MacDowell , N. Florin , A. Buchard , J. Hallett , A. Galindo , G. Jackson , C. S. Adjiman , C. K. Williams , N. Shah , P. Fennell , Energy Environ. Sci. 2010, 3, 1645–1669;

[3c] V. Manovic , J.-P. Charland , J. Blamey , P. S. Fennell , D. Y. Lu , E. J. Anthony , Fuel 2009, 88, 1893–1900;

[3d] DOE, Carbon Capture and Sequestratio Systems Analysis Guidelines, U.S. Department of Energy, **2005**;

[3e] DOE, Hydrogen, Fuel Cells & Infrastructure Technologies Program: Multi-Year Research, Development and Demonstration Plan, **2012**, pp. 3.1-1–3.1-53, https://energy.gov/eere/fuelcells/downloads/fuel-cell-technologies-office-multi-year-research-development-and-22;

[3f] A. MacKenzie , D. L. Granatstein , E. J. Anthony , J. C. Abanades , Energy Fuels 2007, 21, 920–926;

[3g] Z.-S. Li , N.-S. Cai , Y. − y. Huang, H.-j. Han, Energy Fuels **2005**, 19, 1447–1452;

[3h] C. S. Martavaltzi , A. A. Lemonidou , Ind. Eng. Chem. Res. 2008, 47, 9537–9543;

[3i] M. Broda , C. R. Müller , Adv. Mater. 2012, 24, 3059–3064; 22570251 10.1002/adma.201104787

[3j] R. Koirala , G. K. Reddy , P. G. Smirniotis , Energy Fuels 2012, 26, 3103–3109;

[3k] C.-T. Yu , W.-C. Chen , Powder Technol. 2013, 239, 492–498;

[3l] S. F. Wu , Y. Q. Zhu , Ind. Eng. Chem. Res. 2010, 49, 2701–2706;

[3m] M. Zhao , M. Bilton , A. P. Brown , A. M. Cunliffe , E. Dvininov , V. Dupont , T. P. Comyn , S. J. Milne , Energy Fuels 2014, 28, 1275–1283.

[4a] C. C. Dean , J. Blamey , N. H. Florin , M. J. Al-Jeboori , P. S. Fennell , Chem. Eng. Res. Des. 2011, 89, 836–855;

[4b] X. Wang , M. Li , S. Li , H. Wang , S. Wang , X. Ma , Fuel Process. Technol. 2010, 91, 1812–1818.

[5a] H. Yang , Z. Xu , M. Fan , R. Gupta , R. B. Slimane , A. E. Bland , I. Wright , J. Environ. Sci. 2008, 20, 14–27; 10.1016/s1001-0742(08)60002-9 18572517

[5b] K. Nakagawa , T. Ohashi , J. Electrochem. Soc. 1998, 145, 1344–1346;

[5c] B. N. Nair , T. Yamaguchi , H. Kawamura , S. I. Nakao , K. Nakagawa , J. Am. Ceram. Soc. 2004, 87, 68–74;

[5d] B. N. Nair , R. P. Burwood , V. J. Goh , K. Nakagawa , T. Yamaguchi , Prog. Mater. Sci. 2009, 54, 511–541.

[6] M. Khokhani , R. B. Khomane , B. D. Kulkarni , J. Sol-Gel Sci. Technol. 2012, 61, 316–320.

[7a] T. Zhao , E. Ochoa-Fernández , M. Rønning , D. Chen , Chem. Mater. 2007, 19, 3294–3301;

[7b] I. Alcérreca-Corte , E. Fregoso-Israel , H. Pfeiffer , J. Phys. Chem. C 2008, 112, 6520–6525;

[7c] G. G. Santillán-Reyes , H. Pfeiffer , Int. J. Greenhouse Gas Control 2011, 5, 1624–1629;

[7d] L. Martínez-dlCruz , H. Pfeiffer , J. Phys. Chem. C 2012, 116, 9675–9680;

[7e] L. Martínez-dlCruz , H. Pfeiffer , J. Solid State Chem. 2013, 204, 298–304;

[7f] B. Alcántar-Vázquez , Y. Duan , H. Pfeiffer , Ind. Eng. Chem. Res. 2016, 55, 9880–9886.

[8] T. Zhao , M. Rønning , D. Chen , J. Energy Chem. 2013, 22, 387–393.

[9a] W. Nimmo , D. Hind , N. J. Ali , E. Hampartsoumian , S. J. Milne , J. Mater. Sci. 2002, 37, 3381–3387;

[9b] W. Nimmo , N. J. Ali , R. M. Brydson , C. Calvert , E. Hampartsoumian , D. Hind , S. J. Milne , J. Am. Ceram. Soc. 2003, 86, 1474–1480;

[9c] G. L. Messing , S.-C. Zhang , G. V. Jayanthi , J. Am. Ceram. Soc. 1993, 76, 2707–2726.

[10] V. Manovic , E. J. Anthony , Environ. Sci. Technol. 2008, 42, 4170–4174. 18589983 10.1021/es800152s

[11] S. Stendardo , L. K. Andersen , C. Herce , Chem. Eng. J. 2013, 220, 383–394.

[12] J. D. Hancock , J. H. Sharp , J. Am. Ceram. Soc. 1972, 55, 74–77.

[13] A. Khawam , D. R. Flanagan , J. Phys. Chem. B 2006, 110, 17315–17328. 16942065 10.1021/jp062746a

[14] R. Koirala , K. R. Gunugunuri , S. E. Pratsinis , P. G. Smirniotis , J. Phys. Chem. C 2011, 115, 24804–24812.

[15] V. D. A. P. Brown, S. J. Milne, New Approach to Extend Durability of Sorbent Powders for Multicycle High Temperature CO_2_ Capture in Hydrogen, http://gow.epsrc.ac.uk/NGBOViewGrant.aspx?GrantRef=EP/J014702/1, **2012**.

[16] M. Zhao , J. Shi , X. Zhong , S. Tian , J. Blamey , J. Jiang , P. S. Fennell , Energy Environ. Sci. 2014, 7, 3291–3295.

[17] J. A. Mendoza-Nieto , H. Pfeiffer , RSC Adv. 2016, 6, 66579–66588.

[18] G. Ji , M. Z. Memon , H. Zhuo , M. Zhao , Chem. Eng. J. 2017, 313, 646–654.

[19] M. Z. Memon , X. Zhao , V. S. Sikarwar , A. K. Vuppaladadiyam , S. J. Milne , A. P. Brown , J. Li , M. Zhao , Environ. Sci. Technol. 2017, 51, 12–27. 27997129 10.1021/acs.est.6b04992

