# Supplementary material for: Spray‐Dried Sodium Zirconate: A Rapid Absorption Powder for CO2 Capture with Enhanced Cyclic Stability
Source: ChemSusChem. 2017 Apr 13;10(9):2059–67. doi: 10.1002/cssc.201700046 (PMC5516178; doi:10.1002/cssc.201700046)
Supplement: Supplementary file 1 — Supplementary [file CSSC-10-2059-s001.pdf]

CHEMISTRY & SUSTAINABILITY
CHEMSUSCHEM
ENERGY & MATERIALS

Supporting Information

Spray-Dried Sodium Zirconate: A Rapid Absorption Powder for CO_2 Capture with Enhanced Cyclic Stability

Faith Bamiduro,[a] Guozhao Ji,[b] Andy P. Brown,[a] Valerie A. Dupont,[a] Ming Zhao,*[b] and Steven J. Milne*[a]

cssc_201700046_sm_miscellaneous_information.pdf

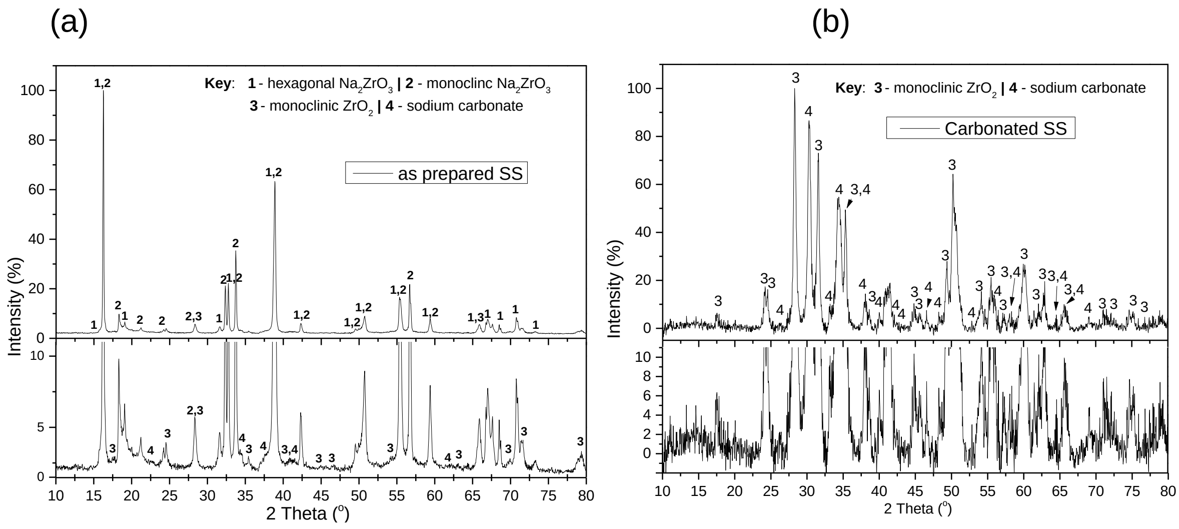

Fig. S1 XRD patterns of conventionally prepared SS powders (a) as –prepared; (b) after 25 min carbonation.

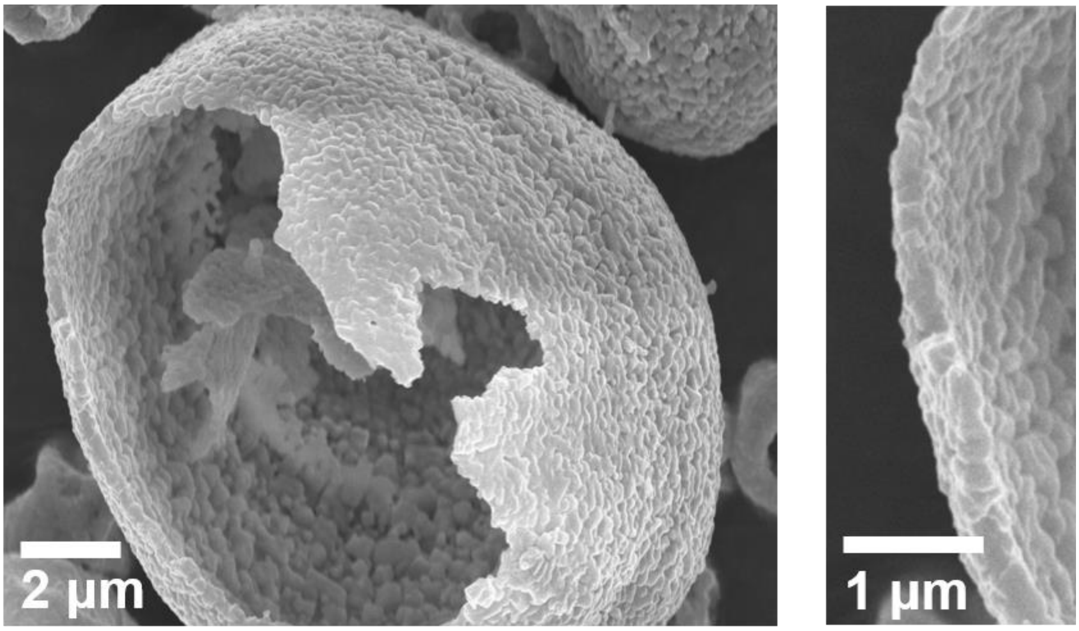

Fig. S2 SEM micrograph revealing the single-crystallite thickness of walls of the SD granules.

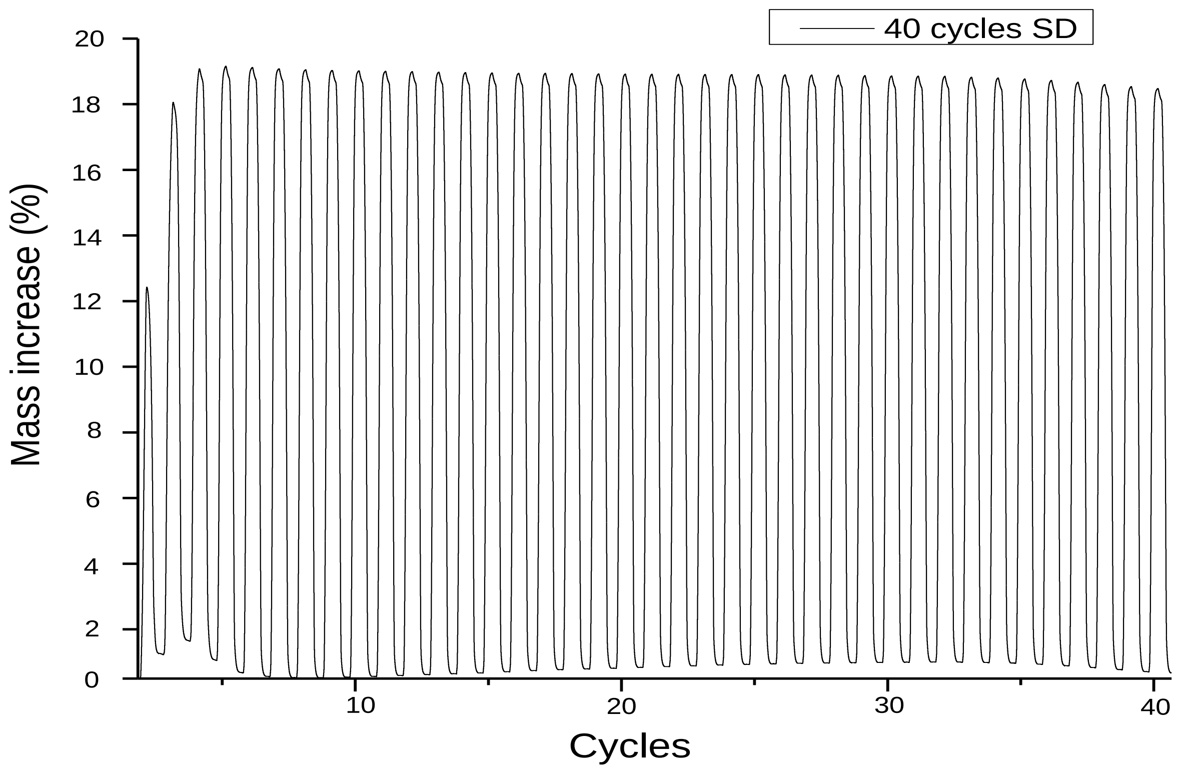

Fig. S3 Cyclic durability (carbonation 5 min in 20% CO_2 at 700 °C, calcination by increasing to 900 °C in N_2 without dwell time).
